# A case of epithelioid osteoblastoma of the thoracic vertebra in a 12-year-old male: a case report and review of literature

**DOI:** 10.1097/MS9.0000000000001430

**Published:** 2023-11-01

**Authors:** Nooshin Zaresharifi, Sahand Karimzadhagh, Zoheir Reihanian, Issa Jahanzad, Mohammad T. Ashoobi

**Affiliations:** a Department of Pathology; b Clinical Research Development Unit of Poursina Hospital; c Department of Neurosurgery, Poursina Hospital; d Department of General Surgery, Guilan University of Medical Sciences; e Pars Hospital, Rasht, Iran

**Keywords:** bone tumor, case report, epithelioid osteoblastoma, osteoblastoma, osteosarcoma

## Abstract

**Introduction and importance:**

Osteoblastoma (OB) is a rare benign bone tumor, representing less than 1% of all bone neoplasms. In contrast to the typical OB, a smaller subset known as ‘epithelioid osteoblastoma (EO)’ exhibits a distinctive inclination for local invasion and recurrence. This rare variant can pose diagnostic challenges, particularly due to its unclear clinical and radiological presentation.

**Case presentation:**

This study details a clinical case of a 12-year-old boy experiencing pain from a lytic bone tumor located in the thoracic vertebrae (T3–T4), initially suggesting malignancy. Following extensive curettage, histopathological analysis confirmed the diagnosis of EO through immunohistochemical staining. Subsequent follow-up at 3 months revealed the absence of no pain or recurrence of the lesion.

**Clinical discussion:**

Distinguishing EO from a malignant tumor requires a multidisciplinary approach, considering clinical, radiographic, and histological features that differentiate the two entities.

**Conclusion:**

The goal of this case presentation is to increase awareness regarding this recurrent tumor variant, which poses diagnostic challenges, particularly in distinguishing it from malignant tumors, including osteosarcoma.

## Introduction

Highlights

- Epithelioid osteoblastoma (EO) is a rare and aggressive type of osteoblastoma due to its tendency to invade locally and its high recurrence rate.
- The diagnosis and treatment of EO require careful assessment of clinical, radiographic, and histopathological findings, as these often exhibit overlapping features with low-grade osteosarcoma (LG-OS) and EO.
- EO is characterized by rounded, well-defined contours, discernible boundaries, and associated expansive growth patterns.
- The most effective treatment strategy for EO entails either extensive curettage or en bloc resection.

Osteoblastomas (OBs) account for 1% of all benign bone tumors^1^. Epithelioid osteoblastoma (EO), specifically, is an uncommon and highly aggressive variant of OB, primarily recognized for its tendency to local invasion and a notably elevated recurrence rate. It derives its name from the distinctive presence of enlarged epithelioid osteoblasts^2^. This bone-forming tumor predominantly affects young adults, with a higher prevalence among males, particularly in patients under the age of 25. It notably tends to manifest in the vertebral column and sacral region^3^.

Clinical manifestations of EO vary depending on the tumor’s location and size. Common symptoms include localized pain, which can be severe and persistent, as well as swelling or palpable masses in the affected area. In cases where the tumor involves the spine, patients may experience neurological deficits such as weakness, numbness, or altered bladder and bowel function due to compression of the spinal cord or nerve roots^4^. Furthermore, some cases may present with pathological fractures if the tumor weakens the bone structure. Given its rarity, recognizing these clinical signs and symptoms is crucial for timely diagnosis and appropriate management of EOs^5^. In radiographical findings, EO on computed tomography (CT) imaging typically appears as a well-defined osteolytic lesion with irregular margins and areas of cortical thinning. Additionally, MRI can provide valuable information about the tumor’s extent, including its relationship to adjacent structures and the spinal cord if it involves the spine^6^. Histopathologically, EO is characterized by an abundance of large epithelioid osteoblasts that encircle the tumor’s periphery. Occasionally, it can exhibit a more aggressive local growth pattern, which may lead to cortical destruction and the development of secondary aneurysmal bone cysts. The diagnosis of OB relies on histological findings, specifically the presence of a bone-forming tumor characterized by trabeculae of remodeled woven bone surrounded by well-developed, plump osteoblasts within a vascularized background^1–3,6^.

The diagnostic challenge associated with OB is further complicated by the spectrum of lesions it can manifest, especially when more severe forms are identified by the presence of epithelioid osteoblasts. Consequently, some pathologists have chosen to classify OB into subcategories, using each distinct feature as a foundation for differentiation. It is imperative for a skilled pathologist to effectively distinguish this specific lesion from conditions like low-grade osteosarcoma (LG-OS) or benign bone tumors such as osteoid osteoma, as their prognosis and clinical implications differ significantly^7^. This report focuses on the case of a 12-year-old boy diagnosed with thoracic spine EO. The patient initially presented with persistent, localized pain in the thoracic spine region, which raised concerns about malignancy.

This work has been reported in line with the SCARE criteria^8^.

## Case presentation

**Figure 1:**
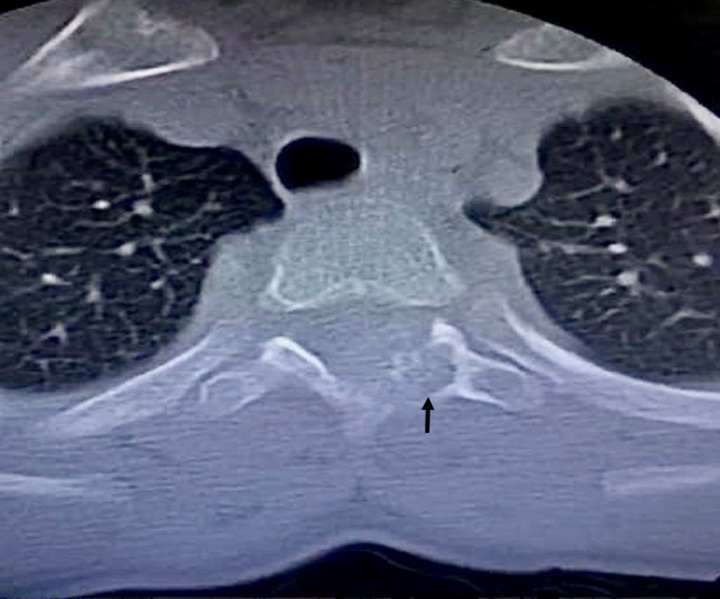
The axial computed tomography scan image of the thorax demonstrated an aggressive lytic lesion (arrow), expanding and scalloping of the posterior pedicles of thoracic vertebrae (T3–T4 level).

**Figure 2:**
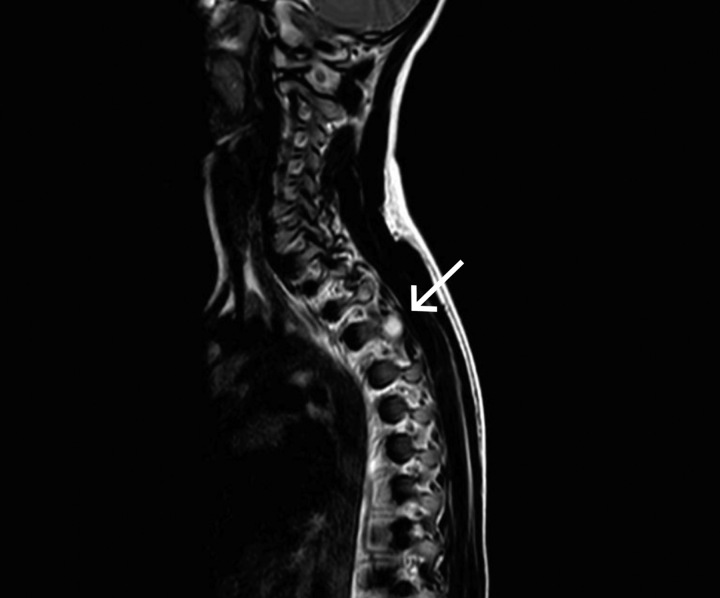
The sagittal T2-weighted MRI image of the thoracic spine reveals a hyperintense lesion measuring 2×2.2×2 cm (arrow).

**Figure 3:**
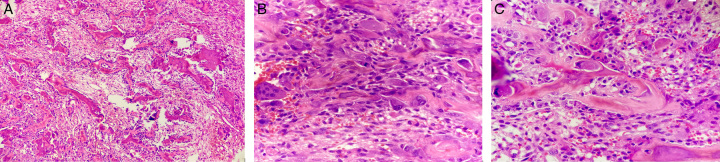
Hematoxylin and eosin (H&E) stain. (A) Inter-anastomosing trabeculae of woven bone, set within loose edematous fibrovascular stroma, with extravasated erythrocytes (×10 magnification). (B, C) The osseous trabeculae are lined by a single layer of large activated epithelioid osteoblasts with eccentric nuclei and prominent nucleoli. Diffusely scattered osteoclast-type multinucleated giant cells. Mitotic activities are sparsely seen, with no atypical forms (×40 magnification).

A 12-year-old boy was referred to the neurosurgery department of our hospital with a 5-month history of persistent moderate pain in the upper thoracic spine, with no associated trauma. On examination, he exhibited a painful, firm, and tender mass in the T3–T4 region of the spine. However, there were no observed weaknesses, sensory impairments, or abnormal reflexes. Notably, the pain exhibited minimal response to non-steroidal anti-inflammatory drugs (NSAIDs). Moreover, the patient denied experiencing fevers, night sweats, or weight loss. The constellation of clinical signs and symptoms prompted further investigation, including radiological imaging. MRI and CT scan revealed an aggressive lytic lesion 2.2×2×2 cm in size that was causing expansion and scalloping of the posterior pedicles of the thoracic vertebrae, specifically at the T3–T4 level (Fig. 1,2). While the first preoperative clinical impression was to rule out a malignant tumor; however, upon a comprehensive retrospective evaluation, distinct features came to light. These notable attributes included the presence of a thin sclerotic rim at the medial margin of the lesion and a tumor growth pattern that displayed expansiveness without infiltrative features. Consequently, these findings led to the confirmation of the alternative histopathological diagnosis. A comprehensive, multidisciplinary preoperative assessment was carried out to ensure the patient’s safety and to plan for the surgical intervention. To establish a definitive diagnosis through histopathological examination, the patient underwent a surgical procedure involving extensive curettage. From the intraoperative perspective, the tumor did not appear to invade adjacent intervertebral discs, nerves, or major blood vessels, with a particular focus on assessing the tumor’s boundaries and its interaction with surrounding anatomical structures to ensure a precise surgical approach. Histopathological examination of the lesion revealed a bone-origin neoplasm characterized by inter-anastomosing trabeculae of woven bone situated within a loose, edematous fibrovascular stroma, with the presence of extravasated erythrocytes. The osseous trabeculae exhibited a distinctive histological pattern characterized by a single layer of prominently activated epithelioid osteoblasts with eccentric nuclei. Diffusely scattered osteoclast-type multinucleated giant cells, in addition to small foci of chondroid differentiation, were also evident (Fig. 3). Immunohistochemistry (IHC) determined the nature and behavior of the tumor by assessing specific markers, including MDM2, β-cadherin, and KI-67. The tumor cells showed only scattered positive staining for MDM2 with no amplification evident (MDM2 mutation is a common molecular finding in osteosarcomas). Tumor staining for β-cadherin demonstrated aberrant nuclear expression as expected, and KI-67 highlighted scattered mitotic activities. In summary, the collective findings from the CT scan and MRI, tumor size (>2 cm), tumor location, and morphological features were consistent with the diagnosis of EO. Importantly, EO carries a considerably more favorable prognosis compared to the primary clinical differential diagnosis, including osteosarcoma or metastatic carcinoma in this case. During the 3-month postoperative follow-up, no complications or signs of tumor recurrence were reported. To ensure ongoing monitoring, we planned to conduct routine radiographs and physical examinations over the subsequent years. This vigilant approach will allow us to assess for any potential recurrence, distant metastasis, or other delayed potential complications that may arise.

## Discussion

Historically, OB was commonly referred to as ‘giant osteoid osteoma,’ underscoring its histopathological resemblance to osteoid osteoma^9^. Nonetheless, the prevailing consensus now asserts that these are distinct pathological entities with differing clinical presentations. OB primarily affects adolescents and young adults, with a mean age of ~20 years. Nevertheless, the more aggressive variants tend to manifest in slightly older patients, with a mean age of around 33 years^3,10,11^. OBs typically exhibit gradual growth and often remain asymptomatic. When symptoms do occur, they commonly appear as localized, dull pain and may involve tenderness^12^. These subtle, nonspecific symptoms often lead to delayed medical evaluation, as evidenced by one study reporting a median symptom duration of 6 months before patients sought clinical attention^13^. Symptomatic patients commonly experience nocturnal discomfort and insufficient relief using pharmacological agents with anti-inflammatory properties. Epithelioid-type OBs may have a higher recurrence rate, leading to the term ‘aggressive osteoblastoma’^14^. However, in accordance with the latest WHO classification in 2020, the term ‘aggressive osteoblastoma’ is no longer recommended, and ‘epithelioid osteoblastoma’ is now the preferred and more accurate term^10^.

Differentiating between EOs and malignancies such as osteosarcoma can pose a challenge due to their histopathological and radiological similarities. Achieving an accurate diagnosis is pivotal when making treatment decisions, as EO does not warrant adjuvant radiation or chemotherapy, in contrast to osteosarcoma^6,15^. The complex radiographic and overlapping histopathological features of EO and LG-OS present significant diagnostic challenges. OB is characterized by rounded, well-defined contours, clear boundaries, and associated expansive growth patterns that lack infiltration into neighboring healthy bone tissue^16^. In contrast, OS exhibits an aggressive periosteal reaction and severe destructive characteristics^16,17^. The absence of pleomorphic atypical nuclei, mitotic activity, and tumor permeation favors the diagnosis of OB over osteosarcoma, as confirmed by previous articles^11,18,19^. Table 1 shows a summary of the radiographical and pathological features of EO in cases reported in the literature. The optimal treatment approach for this specific form of OB typically involves either thorough curettage or en bloc resection, primarily due to the lesion’s inclination toward local invasiveness^29^. The outlook for OB is highly favorable, as the majority of patients achieve a cure through the initial surgical intervention. However, it is important to note that local recurrence is a relatively frequent complication, with rates varying between 15 and 25%^30^. While there is limited supporting evidence regarding the metastatic potential of this pathological lesion, there have been documented instances of patient fatalities associated with the locally aggressive behavior of OB tumors that affect the central neuroaxis^3^. Multiple occurrences of secondary malignant transformation of OB into osteosarcoma have been reported within the literature^23,24,31^. However, the possibility of incorrect identification of the tumor at the onset still exists. Our patient was treated with extensive curettage and is on close follow-up with no evidence of tumor recurrence up till now.

**Table 1:** Summary of the radiographical and pathological appearance of epithelioid osteoblastoma in cases reported since 2015.

References	Location	Radiographic feature	Histopathologic feature	Outcome recurrence
Snow *et al.* ^20^	Hip	The lytic lesion extends into soft tissues with faint mineralization and whorls. Sclerosis surrounds the cortical component, showing a heterogeneous T2 hyperintense signal. Post-contrast imaging reveals peripheral enhancement in larger locules.	Biopsy revealed uniform osteoblasts on tiny trabeculae with abundant cytoplasm and prominent nuclei. Osteoid micronodules formed rosettes. Excised tumor displayed fibrosis, inflammation, lymphoid aggregates, secondary aneurysmal bone cysts, and reactive bone deposition.	No recurrence (6 weeks)
Sharma *et al*.^21^	Parietotemporooccipital bone	CT head (P+C) and CEMRI brain revealed an expansive lesion in the right parietal area, compressing but not infiltrating the adjacent dura mater.	A bony-soft tissue mass was observed with a pericranial outer surface covered by pericranium, and the inner surface (epidural) appeared multilobulated and yellowish-red in color.	Recurrence (15 months)
Rath *et al*.^22^	Navicular bone	The radiograph revealed an osteolytic lesion in the navicular bone with focal granular opacities, and the MRI indicated coalescing areas of bone destruction in the navicular bone.	The excisional biopsy revealed conventional osteoblastoma, along with regions containing osteoid encircled by plump, large epithelioid osteoblasts displaying nuclear pleomorphism, prominent nucleoli, and numerous osteoclastic giant cells.	No recurrence (12 months)
Attiah *et al*.^23^	Temporal bone	Left middle cranial fossa mass with bone erosion, soft tissue extension in sphenoid and temporal bones, periosteal elevation, extending to the facial canal and foramen ovale.	Bony trabeculae in fibrovascular stroma. A single layer of polygonal osteoblasts with round nuclei-rimmed trabeculae. Epithelioid osteoblasts, osteoclasts, and periosteal reactions were presented.	No Recurrence (27 months)
Blank *et al*.^24^	Femur	Large, aggressive lesion near the femur, with mixed lytic and blastic features, a cortical rim on the medial margin. MRI showed a lytic lesion near the femoral neck and a lesser trochanter. Soft tissue mass caused nearby pressure on the iliopsoas and vastus muscles.	Proliferation of uniform large polygonal cells with round nuclei and eosinophilic cytoplasm, forming new woven bone in sheets. No necrosis or high mitotic activity. Positive SATB2 staining for osteoblastic differentiation.	No recurrence (12 months)
Al-Ibraheem *et al*.^25^	Mandible	A lesion showing mixed sclerotic and lytic destruction with outer mandibular cortex damage and submucosal extension.	Irregular bone trabeculae are randomly interconnected, surrounded by plump osteoblasts with abundant cytoplasm and eccentric nuclei that contain prominent nucleoli.	No recurrence (12 months)
Sonnylal *et al*.^1^	Femur	A calcified nodule with a cartilaginous matrix was found in the medullary canal on a CT scan.	A neoplasm with cohesive sheets of osteoblasts, prominent osteoblastic rimming of trabeculae, and fibrovascular stroma.	No recurrence (32 months)
Sharma *et al*.^26^	Acetabulum	CT displayed an expansive lytic lesion with variable-thickness bony septations in the acetabulum, superior pubic ramus, and left iliac blade, accompanied by cortical thinning and an irregular periosteal reaction.	Microscopy showed tumor-forming cell sheets within trabecular spaces. Cells were oval to plasmacytoid with moderate amphophilic vacuolated cytoplasm, moderately pleomorphic nuclei, some binucleated, and a vascular spindle cell stroma with mineralized bone and osteoid.	No recurrence (12 months)
Prabhu *et al*.^27^	Jaw	Radiolucent lesion with radiopacities, cortical bone destruction, and peripheral cortex rim.	The biopsy displayed irregular trabeculae of immature woven bone in a fibrous stroma, resembling a Chinese letter pattern in some areas. Calcifications and plump osteoblasts were present, along with numerous large cells with abundant cytoplasm and eccentric nuclei. Osteoclast-type giant cells resorbed trabeculae, and Pagetoid bone with reversal lines was observed.	Not stated
Schur *et al*.^28^	Cervical Spine	MRI demonstrated a C7-T1 expansile bony lesion with T2 hyperintensity in the surrounding soft tissue	Biopsy displayed neoplastic woven bone trabeculae, large epithelioid osteoblasts with eosinophilic cytoplasm, vesicular nuclei, and prominent nucleoli, confirming aggressive epithelioid osteoblastoma.	Not stated
Rana *et al*.^2^	Maxilla	A lobulated lesion was observed on a CT scan in the right maxilla near the upper inner alveolar margin and molars. The lesion displayed a calcific matrix, ossification, and pleomorphic features.	Large cells with eccentric nuclei and abundant cytoplasm were found alongside osteoid deposits and bony trabeculae. Few giant cells were observed, with no necrosis or abnormal mitotic activity.	Not stated

CEMRI, contrast-enhanced magnetic resonance imaging; CT, computed tomography; P+C, pre-contrast+contrast.

## Conclusion

This case underscores the diagnostic complexities and clinical significance of EO, an uncommon and highly aggressive form of OB. Emphasizing the need for a multidisciplinary approach, this report aims to enhance awareness of EO, aiding healthcare professionals in its accurate identification and timely treatment.

## Ethical approval

This study was approved by the Research Ethics Committee of the Guilan University of Medical Sciences (reference number: IR.GUMS.REC.1402.240).

## Consent

Written informed consent was obtained from the patient’s parents/legal guardian for publication and any accompanying images. A copy of the written consent is available for review on request.

## Consent

Parental Consent for Minors: Written informed consent was obtained from the patient’s parents/legal guardian for publication and any accompanying images. A copy of the written consent is available for review by the Editor-in-Chief of this journal on request.

## Sources of funding

No funding was received.

## Author contribution

All authors contributed equally to this work.

## Conflicts of interest disclosure

There are no conflicts of interest.

## Research registration unique identifying number (UIN)

https://www.researchregistry.com/browse-theregistry#home/registrationdetails/65251d0e857b85002aa669e0/

## Guarantor

Nooshin Zaresharifi and Zoheir Reihanian are the Guarantors for this study and accept full responsibility for the work and the conduct of the study, had access to the data, and controlled the decision to publish.

## Data availability statement

Datasets generated during the current study are available upon reasonable request.

## Provenance and peer review

Not commissioned, externally peer-reviewed.
